# Systems epidemiology and cancer: A review of the National Institutes of Health extramural grant portfolio 2013–2018

**DOI:** 10.1371/journal.pone.0250061

**Published:** 2021-04-15

**Authors:** Marissa M. Shams-White, Rolando Barajas, Roxanne E. Jensen, Melissa Rotunno, Hannah Dueck, Elizabeth M. Ginexi, Scott D. Rogers, Elizabeth M. Gillanders, Leah E. Mechanic

**Affiliations:** 1 Risk Factor Assessment Branch, Epidemiology and Genomics Research Program, Division of Cancer Control and Population Sciences, National Cancer Institute, Bethesda, Maryland, United States of America; 2 Genomics Epidemiology Branch, Epidemiology and Genomics Research Program, Division of Cancer Control and Population Sciences, National Cancer Institute, Bethesda, Maryland, United States of America; 3 Outcomes Research Branch, Healthcare Delivery Research Program, Division of Cancer Control and Population Sciences, National Cancer Institute, Bethesda, Maryland, United States of America; 4 Tumor Biology and Microenvironment Branch, Division of Cancer Biology, National Cancer Institute, Bethesda, Maryland, United States of America; 5 Office of Behavioral and Social Sciences Research, National Institutes of Health, Bethesda, Maryland, United States of America; 6 Epidemiology and Genomics Research Program, Division of Cancer Control and Population Sciences, National Cancer Institute, Bethesda, Maryland, United States of America; Ochsner Clinic Foundation: Ochsner Health System, UNITED STATES

## Abstract

**Objectives:**

Systems epidemiology approaches may lead to a better understanding of the complex and dynamic multi-level constellation of contributors to cancer risk and outcomes and help target interventions. This grant portfolio analysis aimed to describe the National Institutes of Health (NIH) and the National Cancer Institute (NCI) investments in systems epidemiology and to identify gaps in the cancer systems epidemiology portfolio.

**Methods:**

The analysis examined grants funded (2013–2018) through seven NIH systems science Funding Opportunity Announcements (FOAs) as well as cancer-specific systems epidemiology grants funded by NCI during that same time. Study characteristics were extracted from the grant abstracts and specific aims and coded.

**Results:**

Of the 137 grants awarded under the NIH FOAs, 52 (38%) included systems epidemiology. Only five (4%) were focused on cancer systems epidemiology. The NCI-wide search (N = 453 grants) identified 35 grants (8%) that included cancer systems epidemiology in their specific aims. Most of these grants examined epidemiology and surveillance-based questions (60%); fewer addressed clinical care or clinical trials (37%). Fifty-four percent looked at multiple scales within the individual (e.g., cell, tissue, organ), 49% looked beyond the individual (e.g., individual, community, population), and few (9%) included both. Across all grants examined, the systems epidemiology grants primarily focused on discovery or prediction, rather than on impacts of intervention or policy.

**Conclusions:**

The most notable finding was that grants focused on cancer versus other diseases reflected a small percentage of the portfolio, highlighting the need to encourage more cancer systems epidemiology research. Opportunities include encouraging more multiscale research and continuing the support for broad examination of domains in these studies. Finally, the nascent discipline of systems epidemiology could benefit from the creation of standard terminology and definitions to guide future progress.

## Introduction

Cancers are very complex phenotypes and, though many risk factors have been identified and studied through traditional epidemiological research, much of their etiology remains unknown. This is due in part to the relatively siloed focus of many studies on a few risk factors within specific domains (e.g., genetic, behavioral, clinical, or environmental data). Studies are often designed without multilevel approaches, often focused on simple risk factor associations [1]. More research is needed to understand how contributors to cancer risk may be modulated over the lifespan and depend on timing of exposure (e.g., critical windows of susceptibility, cumulative exposure, or acute exposure) [2, 3]. One of the reasons public health interventions may fail is that studies do not account for the dynamic interplay of multiple factors across domains and time [4–6]. Thus, using a more comprehensive, systems-centered approach could allow for a better understanding of disease mechanisms, the contributors to cancer risk and outcomes, and provide insight to better target effective interventions.

A systems approach in science highlights the interconnections and feedback loops between multiple component causes of a disease and the importance of considering how these components interact dynamically over time and at multiple levels of analysis [1, 7, 8]. Systems biology has applied these approaches within complex biological systems with success, such as in studies of gene regulation and interactions between the immune system and the microenvironment, utilizing information from experimental work as well as mathematical and computational modeling [1, 9]. Building on the concepts of systems biology, systems epidemiology is a relatively new approach that can complement traditional epidemiologic approaches to study disease risk and outcomes by incorporating high-dimensional measurements from multiple domains (e.g. environment, genetics, sociodemographic, clinical), while also accounting for complex inter-relationships among multiple risk factors over time [1, 10]. The application of systems approaches in epidemiology can allow for better characterizations of multiple factors influencing complex diseases. For example, computational models can incorporate human genomic, transcriptomic, proteomic, and metabolomic data integrated with global measurements from observational studies to allow epidemiologists to identify contributors to disease and their interactions at multiple levels of analysis [1].

The National Institutes of Health (NIH) has a long history of supporting research using system science approaches. Current high profile NIH programs in this area include: the National Institute of Biomedical Imaging and Bioengineering (NIBIB) Interagency Modeling and Analysis Group (IMAG) [11], the National Institute of General Medical Sciences (NIGMS) Modeling Infectious Disease Agents Study (MIDAS) [12], the NIGMS National Centers for Systems Biology [13], the National Cancer Institute (NCI) Cancer Systems Biology Consortium [14], and the NCI Cancer Intervention and Surveillance Modeling Network (CISNET) [15]. In addition, many NIH funding opportunity announcements (FOAs) in the past decade have encouraged the use of systems science approaches [8], including the “Systems Science and Health in the Behavioral and Social Sciences” which was supported from 2011–2019.

The Division of Cancer Control and Population Sciences at NCI focuses on the research to decrease cancer incidence, morbidity, and mortality by supporting research across the cancer continuum. This portfolio analysis was conducted to determine the extent of NIH-supported research in systems epidemiology, examine the integration of systems epidemiology in cancer control research, and identify gaps in the cancer systems epidemiology portfolio. To accomplish this, we conducted a two-phase grant portfolio analysis. In phase I we sought to characterize the research grants funded under NIH systems science and computational modeling FOAs and to determine the number of systems epidemiology-focused grants. In phase II we aimed to identify and evaluate any additional cancer-focused, systems epidemiology grants in the NCI portfolio.

## Methods

### Phase I: Characterization of grants funded under NIH systems science and computational modeling FOAs

Ten NIH FOAs (fiscal years 2013–2018) were selected that focused on systems science or computational methods. Funded grants from these FOAs were examined for the five years prior to the portfolio analysis start date to both limit the scope and capture the recent emergence of new technologies, omics, and informatic approaches [16, 17]. These “Systems Science and Computational Modeling” FOAs were initially identified in the NIH guide using systems science search terms such as modeling, computational, systems, mathematical, and network [18]. Three FOAs were excluded from review because they did not include systems modeling, systems science, multilevel, dynamics, or integration in the FOA descriptions. The remaining seven FOAs and characteristics of the funded grants (N = 137) identified in the NIH administrative grants database (the Information for Management Planning Analysis and Coordination known as IMPAC II) over this five-year period are provided in Table 1. These grants may also be found by searching NIH RePORTER (reporter.nih.gov) following instructions provided (S1 Appendix).

**Table 1:** Funding opportunity announcements selected for inclusion in phase I of the portfolio analysis (N = 137).

FOA #	ICs/Offices on RFA	FOA title	Number Funded
PAR-18-331	NIMHD, NCI, NHLBI, NIA, NIDCD, NIDA, NIMH, NLM, ODP, OBSSR, NIAAA, NIBIB	Simulation Modeling and Systems Science to Address Health Disparities (R01Clinical Trial Not Allowed)	1
PAR-16-131	NCI	Emerging Questions in Cancer Systems Biology (U01)	10
RFA-HL-18-020	NHLBI	Integrative Computational Biology for Analysis of NHLBI TOPMed Data (R01)	7
PAR-15-085 PAR-11-203	NIBIB, NCI, NHGRI, NIA, NIAAA, NIAMS, NICHD, NIDA, NIEHS, OBSSR, NHLBI, NCCIH, ARO, DOE, FDA, NASA, NSF, ONR	Predictive Multiscale Models for Biomedical, Biological, Behavioral, Environmental and Clinical Research (U01)	61
PAR-15-048 PAR-15-047 PAR-11-315 PAR-11-314	OBSSR, NCI, NIA, NIAAA, NIBIB, NICHD, NIDCR, NIEHS, NIMH, NINR, ODP, NIGMS	Systems Science and Health in the Behavioral and Social Sciences (R01, R21)	38
PAR-17-267 PA-16-107 RFA-GM-14-007	NIGMS, NIAID	Modeling of Infectious Disease Agent Study Research Projects (R01)	15
PAR-13-081	NCI, NIAAA	Bridging the Gap Between Cancer Mechanism and Population Science (U01)	5
		**Total:**	**137**

ARO, Association for Research in Otolaryngology; DOE, Department of Energy; FDA, Food and Drug Administration; NASA, National Aeronautics and Space Administration; NCCIH, National Center for Complementary and Integrative Health; NCI, National Cancer Institute; NHLBI, National Heart, Lung, and Blood Institute; NHRGI, National Heart, Lung, and Blood Institute; NIA, National Institute on Aging; NIAAA, National Institute on Alcohol Abuse and Alcoholism; NIAID, National Institute of Allergy and Infectious Diseases; NIAMS, National Institute of Arthritis and Musculoskeletal and Skin Diseases; NIBIB, National Institute of Biomedical Imaging and Bioengineering; NICHD, National Institute of Child Health and Human Development; NIDA, National Institute on Drug Abuse; NIDCD, National Institute on Deafness and Other Communication Disorders; NIDCR, National Institute of Dental and Craniofacial Research; NIEHS, National Institute of Environmental Health Sciences; NIGMS, National Institute of General Medical Sciences; NIMH, National Institute of Mental Health; NIMHD, National Institute on Minority Health and Health Disparities; NINR, National Institute of Nursing Research; NLM, National Library of Medicine; NSF, National Science Foundation; OBSSR, Office of Behavioral and Social Sciences Research; ODP, Office of Disease Prevention; ONR, Office of Nutrition Research; PAR, Program Announcement Reviewed by an Institution

We characterized the funded grants solicited from these FOAs based on their abstracts and specific aims. Reviewers extracted detailed information from the grants using the terms and definitions found in Table 2. The definition for systems epidemiology was adapted from Dammann et al. [1]: an epidemiologic approach to study disease risk and outcomes that incorporates high-dimensional measurements from multiple domains (e.g. environment, genetics, sociodemographic, clinical), inter-relationships between risk factors, and changes over time. We defined domains as general categories of risk factors. Domains look beyond scales or levels (e.g., within the person, interpersonal, environmental) to further disaggregate exposures by context (e.g., the individual level was stratified into the domains demographics, biology and genomics, and individual exposures). Additionally, the concept of dynamism was adapted from Luke et al. [19]. However, a few assumptions were made given the limited information provided in the specific aims. The mention of multiple domains within a specific aim(s) was assumed to meet the criteria of integration across domains, even when the integration methodology was not clearly or explicitly stated. Similarly, if authors included verbiage identifying two or more time points within an aim(s), it was assumed to meet the definition of dynamism. The remaining terms and categories in Table 2 were iteratively created and defined by our portfolio analysis team to clarify multiscale within versus beyond the person, the research content area(s), outcome(s) of interest, and purposes(s) of the studies. Given that grants can have multiple aims, and aims can have multiple purposes, many of the categorizations were not mutually exclusive (Table 2).

**Table 2:** Definitions of specific aims characteristics used during extractions.

Term	Definition
Systems epidemiology	An epidemiologic approach to study disease risk and outcomes that incorporates high-dimensional measurements from multiple domains (e.g. environment, genetics, sociodemographic, clinical), inter-relationships between risk factors, and changes over time^a^
Multiscale approach	
Within person	Combined models at multiple scales in within the person (may also be referred to as multilevel) (e.g., gene, cell, and tissue)
Beyond the person	Combined models at multiple scales beyond the person (may also be referred to as multilevel) (e.g., individual exposure, community exposure)
Dynamism	Included multiple time points or consider changes over time
Measurement domain	Selected the term(s) that apply from the list below to describe the domain of the data analyzed. Extractors included any domain with at least one measurement or variable. Simulated data were categorized according to the domain being simulated. Please note: If it only appears to be a covariate the model adjusted, it was excluded.
Individual exposures	Exposure measure for an individual (e.g. diet, smoking, physical activity, sleep, stress, other behavioral factors, personal relationships, biomarkers of exposure (including nutritional markers, microbiome)
Group/community exposures	Exposure for a group of people or a community (e.g. food access, tobacco environment, environmental toxins, pollution, social opportunities (e.g. opportunities to meet new people in denser populations)
Biology and genomics	E.g. sequencing, transcriptomics, germline variation, genotype, genome-wide association study (GWAS)
Demographics	E.g. socioeconomic status, race, gender
Clinical	E.g. electronic health records, medical records, diagnosis, co-morbidities, treatment, Medicare billing data, clinical measures (e.g., blood pressure, cholesterol, triglycerides, HbA1c)
Imaging	Utilized imaging techniques
Other	*(Extractor provided specifics if selected)*
Integration across domains	At least two measurement domains were analyzed together. Adjustment for confounding was not be considered integration.
Cancer-focused study	Included studies focused on either cancer or cancer-related behaviors/risk factors (e.g., smoking)
Research content area	Selected the term(s) that apply from the list below to describe research content area.
Epidemiology and surveillance	Studies of the distribution and determinants of health-related states or events (including disease) and application of this study to the control of diseases and other health problems. Note: Includes molecular epidemiology
Basic biology	Research aimed at providing mechanistic insights, primarily includes research with in vitro models or biological specimens. Note: mechanistic focus; excludes molecular epidemiology
Clinical care/Clinical trials	Research about the application of clinical care, health care delivery, or clinical trials research (E.g. studies involving health care delivery, pharmaceutical trials)
Behavioral	Research focused on the observable actions of individuals or groups and to mental phenomena such as knowledge, attitudes, beliefs, motivations, perceptions, cognitions, and emotions^b^
Other	*(Extractor provided specifics if selected)*
Outcome of interest	Selected the term(s) that apply from the list below to describe the topic(s) under study. Multiple categories may apply.
Biological insight/Mechanism	Looked at the mechanisms or the function of healthy/diseased tissue
Disease risk	Looked at the risk of disease or cancer
Disease-related outcomes	Looked at outcomes of disease (included response to therapy)
Other	Could include health outcomes not directly related to disease (e.g., quality of life, symptom management), biomarker measure(s) *(Extractor provided specifics if selected)*
Purpose	Selected the term(s) that apply from the list below to describe the goal(s) of the study. Multiple categories may apply.
Discovery/Prediction	To gain insight or knowledge on something previously unknown (e.g., identifying mechanisms, new risk factors) and/or to predict an outcome using identified risk factors
Intervention/Policy	To examine the effects of policy changes (e.g., effects of changes in soda tax policy) and/or inform the design or forecasting effects of interventions

^a^ Adapted from Dammann et al. [1]

^b^ Definition adopted from the Office of Behavioral Sciences and Social Sciences research [20]

At the beginning of extraction, all reviewers (MSW, RB, RJ, MR, EG, LM) reviewed and extracted nine percent of the 137 grants (N = 12) to ensure consistency and similar interpretation of the data extraction template and term definitions by all reviewers. The remaining grants (N = 125) were divided into batches of 10–15 and double-extracted, whereby one reviewer extracted the information and a second reviewed and confirmed the extractions, to minimize extraction errors and reduce reviewer bias. The reviewers paired for extractions were rotated to maintain consistency in extractions across groups. Any disagreements were resolved through discussion amongst reviewer pairs, and any remaining discrepancies resolved via group consensus.

### Phase II: Identification of cancer-focused, systems epidemiology grants through NCI-specific grants search

Unlike phase I, where the focus was describing grants funded from the NIH FOAs, the focus in phase II was to identify and describe grants identified as “cancer systems-epidemiology,” regardless of what funding announcement was used. The decision to narrow phase II’s focus to cancer and epidemiology was based on our interest in an analysis to inform the NCI-specific cancer epidemiology research portfolio. In January 2019, the NIH grants database containing information on NCI extramural research projects was searched using NCI’s Portfolio Management Application (PMA) system to search IMPAC II to identify cancer-focused, systems epidemiology grants funded between fiscal years 2013–2018. The search strategy included performing a text search of the titles, abstracts, and specific aims of funded grants using variations of the following search terms: agent-based model; biomolecular model; computational model; machine learning; multilevel; multiscale model; network analysis; network-based; simulation model; systems-based; systems dynamics; systems epidemiology; systems model; and systems science. The project summaries of these grants may be viewed in NIH RePORTER using methods provided (S1 Appendix). Any duplicate results between the two databases were removed. Identified grants (N = 453) were first screened (by MSW and LEM) to only include grants with human subjects and cancer outcomes or cancer-relevant behaviors (e.g., smoking) (N = 307). Cancer-focused grants extracted in phase I that appeared in phase II (N = 3) were not re-extracted; the specific aims from two grants from phase I did not include any of the search terms and thus was not included. The included grants (N = 145) were separated into batches of 15–30 and reviewed similarly to phase I (i.e., one extractor, one reviewer); reviewers only extracted the characteristics in Table 2 for those grants that met the working definition of systems-epidemiology (N = 35). Discrepancies were resolved by MSW and LEM where appropriate.

## Results

The first section of the results describe the grants identified in phase I and the phase II cancer-specific grants are described in the second section. Characteristics of the grants analyzed are described in detail in Tables 3 and 4, respectively.

**Table 3:** Characteristics of phase I grants: Overall and systems epidemiology specific aims^a^.

Characteristic (N (%))	Phase I: Overall	Phase I: Systems Epidemiology
	(N = 137)	(N = 52)
Multiscale approach		
Within person	84 (61%)	18 (35%)
Beyond the person	57 (42%)	44 (85%)
Cancer-focused	20 (15%)	5 (10%)
Measurement domains		
Individual exposure(s)	51 (37%)	36 (69%)
Group/community exposure(s)	50 (37%)	37 (71%)
Biology and genomics	91 (66%)	21 (40%)
Demographics	29 (21%)	24 (46%)
Clinical	43 (31%)	24 (46%)
Imaging	20 (15%)	4 (8%)
Other	3 (2%)	1 (2%)
Research content area		
Epidemiology & surveillance	56 (41%)	40 (77%)
Basic biology	74 (54%)	10 (19%)
Clinical care/Clinical trials	23 (17%)	15 (29%)
Behavioral	24 (18%)	16 (31%)
Other	2 (2%)	0 (0%)
Outcome of interest		
Biological insight/Mechanism	63 (46%)	4 (8%)
Disease risk	44 (32%)	30 (58%)
Disease-related outcomes	42 (31%)	17 (33%)
Other	15 (11%)	8 (15%)
Purpose		
Discovery/Prediction	122 (89%)	44 (85%)
Intervention/Policy	33 (24%)	16 (31%)

^a^ The characteristics listed are defined as described in the Methods

**Table 4:** Characteristics of phase II cancer systems epidemiology grants^a^.

Characteristic (N (%))	Phase II: Cancer Systems Epidemiology (N = 35)
Year funded	
2013	2 (6%)
2014	3 (9%)
2015	4 (11%)
2016	7 (20%)
2017	8 (23%)
2018	11 (31%)
Multiscale approach	
Within person	20 (54%)
Beyond the person	18 (49%)
Measurement domains	
Individual exposure(s)	20 (56%)
Group/community exposure(s)	17 (47%)
Biology and genomics	21 (58%)
Demographics	10 (28%)
Clinical	21 (58%)
Imaging	9 (25%)
Other	3 (8%)
Research Content area	
Epidemiology & surveillance	21 (60%)
Basic biology	7 (20%)
Clinical care/clinical trials	13 (37%)
Behavioral	4 (11%)
Other	0 (0%)
Outcome of interest	
Biological insight/Mechanism	2 (6%)
Disease risk	9 (26%)
Disease-related outcomes	18 (51%)
Other	9 (26%)
Purpose	
Discovery/Prediction	32 (91%)
Intervention/Policy	6 (17%)

^a^ The characteristics listed are defined as described in the Methods

### Overview of phase I: Characterization of grants funded under NIH systems science and modeling FOAs

#### Phase I grants overall

The Systems Science and Computational Modeling FOAs were supported across several institutes at NIH and awarded 137 grants in fiscal years 2013–2018. Of these, only 84 (61%) and 57 (42%) utilized a multiscale approach within and/or beyond the person, respectively (Table 3). Dynamism was included in 115 (84%) of the grants. One hundred and one (74%) grants included integration across domains, with biology and genomics as the most common measurement domain (N = 91, 66%). The most common research content area was basic biology (N = 74, 54%), while the main outcomes of interest were biological insight/mechanism (N = 63, 46%) and disease risk (N = 44, 32%). The primary purpose for 122 (89%) of the grants was discovery and/or prediction (Table 3).

#### Phase I systems epidemiology grants

**Fig 1:**
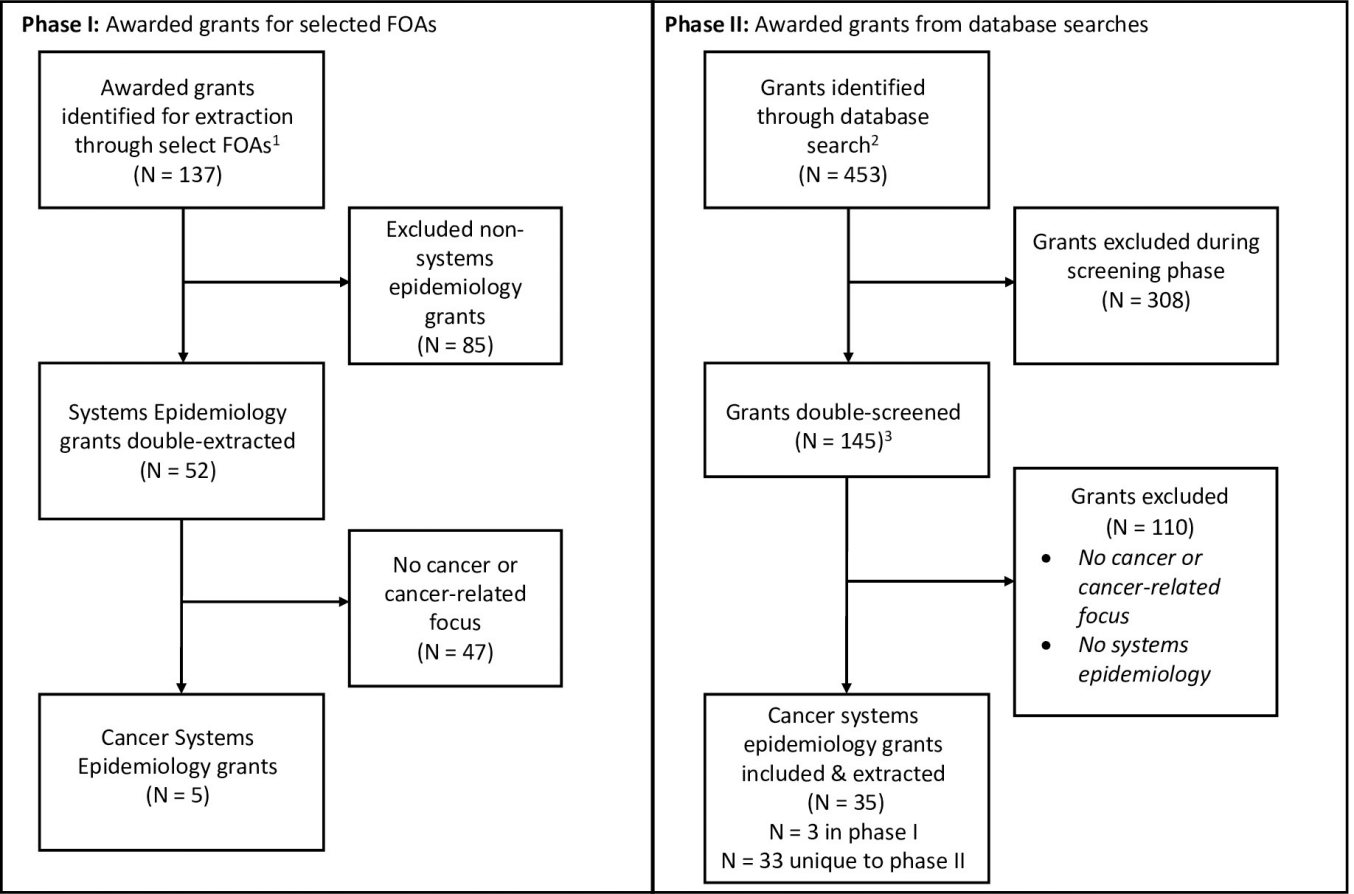
PRISMA flow diagram. FOAs, funding opportunity announcements ^1^ Grants were included if they were awarded between 2013–2018 through the select FOAs. ^2^ The Portfolio Management Application (PMA) database was used for the specific aims search. ^3^ Of the 146 Specific Aims, three were already extracted in phase I and thus not re-extracted in phase II.

Of the 137 grants awarded under these FOAs, 52 (38%) were identified as focused on systems epidemiology, of which five (10%) were categorized as cancer systems epidemiology grants (Table 3, Fig 1, S1 Table). Compared to phase I grants overall, a higher proportion of the phase I systems epidemiology grants were characterized as including a multiscale approach within (N = 18, 35%) and/or beyond the person (N = 44, 85%). Only 21 (40%) grants included the biology and genomics domains, as most focused on individual (N = 36, 69%) and group/community exposure (N = 37, 71%) domains. The most common research content area was epidemiology and surveillance (N = 40, 77%), while the primary outcomes of interest focused on disease risk (N = 30, 58%) and disease-related outcomes (N = 17, 33%). Similar to phase I grants overall, the systems epidemiology grants primarily focused on discovery and/or prediction (N = 44, 85%).

### Overview of phase II: Identification of grants through the NCI-specific grant search

Our database search for phase II initially yielded 453 grants. After review of the grant aims for inclusion of epidemiology and cancer, 145 grants were reviewed by paired reviewers, of which 35 grants were identified and coded as including cancer systems epidemiology (Fig 1, S2 Table). This represents less than 1% of grants funded at NCI during the same time period. The number of cancer systems epidemiology grants funded increased by year (Table 4), with the most funded in 2018 (N = 11, 31%). Approximately half of the grants combined models at multiple scales in different domains within the person (54%) and/or beyond the person (49%); few grants (9%) included both. Exploring the domains, the grants primarily analyzed biological and genomic data (N = 21, 58%), clinical data (N = 21, 58%), individual exposure data (N = 20, 56%), and group/community exposure data (N = 17, 47%). Forty percent of the grants included two domains and 60% included three or more domains: individual and community/group exposure domains commonly appeared together (N = 15 grants), as did the combination of biology/genomics and clinical domains (N = 13 grants). The main research content areas were epidemiology and surveillance (60%) and clinical care/clinical trials (37%), with the main outcome of interest being disease-related outcomes (51%). The primary purpose of the phase II grants was discovery and/or prediction (91%) (Table 4).

## Discussion

Systems approaches may lead to a better understanding of the complex and dynamic multi-level constellation of contributors to both cancer risk and outcomes and may more precisely inform and target interventions [21]. The goals of this grant portfolio analysis were to describe NIH and NCI investments in systems epidemiology and to identify gaps specific to the cancer systems epidemiology portfolio. The analysis examined grants funded (2013–2018) through several NIH systems science FOAs (phase I) as well as cancer-specific systems epidemiology grants received outside of these FOAs and funded by NCI during that same time (phase II).

Overall, one of the most notable findings of the present analysis was that systems science grants focused specifically on cancer compared to the overall cancer portfolio was relatively small. Characteristically speaking, the majority of the overall NIH systems science portfolio over this time period was represented by: within person approaches, biology and genomics measurement domains, basic biology or epidemiology and surveillance research content areas, and with outcomes focused on mechanistic insights for the purposes of discovery or prediction (see Tables 3 and 4 for a summary). By contrast, the grants focusing on use of system science in epidemiologic research included more research focused beyond the person and focused more often on disease risk/disease related outcomes for the purposes of discovery or prediction.

The fact that the majority of awarded grants from the selected NIH Systems Science and Computational Modeling FOAs focused on basic biology, genomics, or mechanistic applications is a finding consistent with the maturity of the systems biology field [20, 22]. Thirty-eight percent of the phase I grants also included a systems epidemiology approach, suggesting that these FOAs attracted and supported population-based applications. Even though NCI participated in five of the seven FOAs, very few systems epidemiology grants included cancer populations, cancer-specific risks, or cancer-related outcomes. Results were consistent in phase II, where cancer systems epidemiology grants made up less than one percent of grants funded by NCI in the same time period.

The reason for the small number of systems epidemiology grants involving cancer is unknown. It is possible that more extensive characterization (e.g., repeated surveys, electronic health record linkages, physical measurements, biospecimens) over time is needed to support cancer systems epidemiology research. Several additional challenges to conducting systems epidemiology included transdisciplinary research, data sharing, and training needs, as described in detail elsewhere [10].

Notably, the number funded from NCI increased annually. Recent advances may have contributed to this growth, including the ability to link datasets; the growing availability of big data and usage of both real and simulated data; and more sophisticated machine learning systems for predictive modeling [23, 24]. Both the support of population-based studies in the Systems Science and Computational Modeling FOAs (phase I) and the small number of cancer systems epidemiology grants in the NCI portfolio (phase II) suggest an opportunity exists for more targeted, cancer-specific FOAs to facilitate further growth.

Our definition of systems epidemiology focused on disaggregating exposures by context via defined domains and did not explicitly include a requirement for research to cross multiple scales. However, complex diseases are influenced by factors on multiple scales and, thus, their integration into models can improve research from discovery and prediction through intervention and policy [1, 19]. In our review of the grants, we noted that biology-focused grants often considered multiple scales within the person (e.g., cell, tissue, organ), while epidemiology-based grants often considered multiple scales beyond the person (e.g., individual, community, population). Just over half of the cancer systems epidemiology grants included multiscale approaches within the individual and/or beyond the individual. This highlights a future opportunity in cancer systems epidemiology research to encourage more multiscale studies

It was encouraging to see that multiple domains (> two domains) were included in many systems epidemiology grants in phase II. We noticed that individual and group/community exposure domains (e.g., impact of both emotions and peer behaviors on smoking cessation outcomes) were commonly included and often together. Genomics and clinical domains were also often grouped together (e.g., impact of BRCA testing and cancer prevention interventions collected via electronic health records on cancer incidence and mortality). There remains an opportunity for a broader inclusion of domains in systems epidemiology research. As previously mentioned, the linking of datasets and availability of electronic health records and genomics data may lead to more diversity and cross-domain work in future grant submissions.

One overarching challenge of this portfolio analysis that applies to the current systems epidemiology field was the lack of universal nomenclature. There is a need for the widespread use of standard terminology and definitions–or “branding”–in this relatively nascent field to promote systems epidemiology research. Part of the challenge of succinctly defining systems epidemiology is that it is a research approach rather than a single method [1]. Though our definition of systems epidemiology closely resembles that of Dammann et al. [1], it differs from other researchers’ definitions [2, 4, 5]. The lack of standard terminology may have impacted our search and caused us to miss relevant grants, especially if none of the grants included the term “systems epidemiology.” Moreover, definitions for systems approaches has evolved over time, and researchers may have conducted systems research without using the included terminology. This explains why two of the five cancer systems epidemiology grants from phase I was not captured in phase II. However, given our extensive list of search terms and the similar distribution of characteristics in phases I and II, we do not believe the lack of nomenclature standards significantly impacted our overall results or interpretation.

The main limitation of this study is that we only extracted data from the grant abstracts and specific aims; full grants were not reviewed. If systems epidemiology was not clearly represented in projects’ abstracts and specific aims, it was not included. Therefore, our results may not reflect the totality of NIH funded grants in systems epidemiology or cancer systems epidemiology, respectively. Though we accounted for this limitation by being broad-minded in the interpretation of our definitions, this may conversely overestimate the extent of work being done in the portfolio. For example, an assumption was made that if a grant mentioned more than one time point in a model, dynamism was present. Similarly, if more than one domain was mentioned within an aim, we assumed the domains were integrated.

## Conclusions

A wide breath of opportunities for future research involving cancer systems epidemiology were identified by this portfolio review, including encouraging more multiscale research and continuing the support for broad examination of domains in these studies. Innovations in data science, medical informatics, electronic health records, mobile and wearable technologies, and new methods to link and analyze big data are creating a potentially ideal environment for the advancement of systems epidemiology as a complementary approach to traditional epidemiology. Keys to success will be the ability to integrate complex, multiscale data from a wide range of sources, and the fostering of interdisciplinary collaborations that will allow for the integration of expertise from different disciplines. The voluminous information available now for epidemiological inquiry in cancer can come from diverse data sources and will benefit from systems approaches to accurately model this complexity. Finally, the creation of standard terminology and definitions could prove instrumental in guiding future funding opportunities and supporting dialogue among the growing community of systems epidemiology scientists.

## Supporting information

S1 Table Phase I systems epidemiology grants awarded from selected funding opportunities (Fiscal Years 2013–2018). (XLSX)

S2 Table Phase II cancer-focused, systems epidemiology grants (Fiscal Years 2013–2018). (XLSX)

S1 Appendix Replication of findings using NIH RePORTER. (DOCX)
