# Supplementary material for: Systems epidemiology and cancer: A review of the National Institutes of Health extramural grant portfolio 2013–2018
Source: PLoS One. 2021 Apr 15;16(4):e0250061. doi: 10.1371/journal.pone.0250061 (PMC8049352; doi:10.1371/journal.pone.0250061)
Supplement: S1 Appendix — (DOCX) [file pone.0250061.s003.docx]

## **S1. Appendix: Replication of Findings Using NIH RePORTER**

## **Phase I:** **Characterization of grants funded under NIH Systems Science and Computational Modeling FOAs**

Grants awarded by the ten FOAs listed in Table 1, can be identified in NIH RePORTER (<https://reporter.nih.gov/>) using the following procedures.

1. Go to “Advanced Projects Search”
2. Under “Fiscal Year”, select: 2013, 2014, 2015, 2016, 2017, and 2018.
3. Expand search options by selecting “Click here to view All Search Fields”
4. Under FOA, enter: PAR-18-331; PAR-16-131; RFA-HL-18-020; PAR-15-085; PAR-11-203; PAR-15-048; PAR-15-047; PAR-11-315; PAR-11-314; PAR-17-267; PA-16-107; RFA-GM-14-007; PAR-13-081
5. Under “Award Type”, select “Competing Renewal” and “New”
6. Click box for “Exclude Subprojects”

## The specific aims for these grants were reviewed and data was extracted and summarized as described in “Methods”.

## **Phase II: Identification of cancer-focused, systems epidemiology grants through NCI-specific grants search**

## Project summaries for grants identified using the term search in Phase II, can be viewed in NIH RePORTER (<https://reporter.nih.gov/>) using the following procedures.

1. Go to “Advanced Projects Search”
2. Under “Fiscal Year”, select: 2013, 2014, 2015, 2016, 2017, and 2018.
3. Expand search options by selecting “Click here to view All Search Fields”
4. Click box for “Exclude Subprojects”
5. Under “Project Number/Application ID” enter: 1F30CA189793-01;1F30CA196020-01;1F30CA203154-01A1;1F30CA203220-01;1F30CA206291-01A1;1F30CA206333-01A1;1F30CA210329-01;1F30CA213728-01;1F30CA213737-01;1F30CA213876-01;1F30CA224687-01A1;1F30CA224968-01A1;1F30CA225046-01;1F31CA171789-01A1;1F31CA176910-01A1;1F31CA177203-01;1F31CA180477-01A1;1F31CA189794-01;1F31CA192478-01;1F31CA200242-01;1F31CA200266-01;1F31CA200322-01A1;1F31CA206334-01;1F31CA206426-01A1;1F31CA210399-01A1;1F31CA210607-01;1F31CA210616-01;1F31CA210622-01;1F31CA213744-01;1F31CA213813-01A1;1F31CA214029-01;1F31CA221061-01A1;1F31CA228331-01A1;1F31CA232465-01;1F32CA165657-01A1;1F32CA168497-01A1;1F32CA174221-01;1F32CA189246-01;1F32CA196149-01A1;1F32CA213966-01;1F32CA214030-01;1F32CA216937-01A1;1F32CA233203-01;1F99CA212231-01;1F99CA212439-01;1F99CA212457-01;1F99CA222722-01;1K01CA175239-01A1;1K01CA184288-01;1K01CA193667-01;1K01CA193918-01A1;1K01CA204456-01A1;1K01CA226355-01;1K01CA229996-01;1K01CA234226-01;1K01CA234453-01;1K07CA172677-01A1;1K07CA177900-01A1;1K07CA178331-01;1K07CA180782-01A1;1K07CA187403-01A1;1K07CA190673-01;1K07CA211955-01A1;1K07CA211971-01A1;1K07CA218377-01A1;1K07CA222343-01A1;1K08CA222676-01A1;1K08CA234326-01;1K08CA234458-01;1K22CA188169-01;1K22CA204439-01A1;1K22CA216318-01;1K24CA194251-01;1K24CA208132-01;1K24CA212294-01;1K25CA168936-01A1;1K25CA204526-01;1K25CA204599-01;1K99CA172360-01;1K99CA175290-01;1K99CA190890-01A1;1K99CA191021-01A1;1K99CA191093-01;1K99CA191152-01;1K99CA194163-01;1K99CA207730-01A1;1K99CA207744-01;1K99CA207871-01;1K99CA218891-01A1;1K99CA218900-01A1;1K99CA222554-01;1K99CA226396-01;1K99CA230195-01;1P01CA225597-01;1R00CA168997-01;1R01CA160911-01A1;1R01CA166011-01A1;1R01CA168677-01A1;1R01CA168795-01A1;1R01CA169122-01A1;1R01CA172211-01A1;1R01CA172279-01A1;1R01CA172638-01A1;1R01CA172986-01A1;1R01CA174468-01;1R01CA174785-01A1;1R01CA175112-01A1;1R01CA175747-01A1;1R01CA176078-01A1;1R01CA178393-01A1;1R01CA178875-01A1;1R01CA178941-01;1R01CA179902-01A1;1R01CA179977-01;1R01CA179991-01;1R01CA180149-01;1R01CA180776-01;1R01CA181664-01A1;1R01CA182514-01A1;1R01CA184510-01A1;1R01CA184772-01;1R01CA185137-01;1R01CA185207-01;1R01CA185486-01;1R01CA186714-01;1R01CA187027-01A1;1R01CA188813-01A1;1R01CA190329-01A1;1R01CA192345-01;1R01CA192402-01A1;1R01CA192652-01A1;1R01CA192838-01;1R01CA193650-01;1R01CA193730-01A1;1R01CA194321-01A1;1R01CA194461-01;1R01CA194697-01;1R01CA194783-01;1R01CA196018-01;1R01CA196631-01A1;1R01CA196667-01;1R01CA196701-01;1R01CA197000-01A1;1R01CA197059-01;1R01CA197178-01A1;1R01CA197205-01;1R01CA197398-01;1R01CA197491-01A1;1R01CA198121-01A1;1R01CA198915-01;1R01CA198971-01;1R01CA199673-01;1R01CA199996-01;1R01CA200690-01A1;1R01CA200718-01;1R01CA200845-01A1;1R01CA200859-01;1R01CA201358-01A1;1R01CA201415-01A1;1R01CA202756-01A1;1R01CA202956-01A1;1R01CA203984-01;1R01CA204004-01A1;1R01CA204070-01;1R01CA204261-01A1;1R01CA204320-01A1;1R01CA206100-01A1;1R01CA206180-01A1;1R01CA206279-01A1;1R01CA206877-01;1R01CA207026-01A1;1R01CA207260-01A1;1R01CA207361-01A1;1R01CA207401-01A1;1R01CA207456-01;1R01CA207540-01A1;1R01CA208148-01;1R01CA208179-01A1;1R01CA208517-01;1R01CA208851-01A1;1R01CA210806-01A1;1R01CA211048-01;1R01CA211141-01A1;1R01CA211224-01A1;1R01CA211323-01A1;1R01CA211625-01;1R01CA211711-01;1R01CA211723-01A1;1R01CA212086-01A1;1R01CA213466-01;1R01CA213990-01;1R01CA214085-01;1R01CA214427-01A1;1R01CA214428-01A1;1R01CA214526-01;1R01CA214530-01;1R01CA214608-01;1R01CA214825-01;1R01CA214981-01A1;1R01CA215155-01A1;1R01CA215318-01;1R01CA215418-01A1;1R01CA215420-01A1;1R01CA215498-01A1;1R01CA215574-01A1;1R01CA216863-01;1R01CA217165-01A1;1R01CA217889-01A1;1R01CA218094-01A1;1R01CA218144-01;1R01CA218187-01A1;1R01CA218188-01A1;1R01CA218357-01A1;1R01CA218405-01A1;1R01CA218664-01A1;1R01CA218668-01A1;1R01CA218739-01A1;1R01CA218923-01A1;1R01CA220591-01A1;1R01CA220693-01;1R01CA221260-01A1;1R01CA221916-01A1;1R01CA222007-01A1;1R01CA222064-01A1;1R01CA222218-01A1;1R01CA222469-01;1R01CA222831-01A1;1R01CA222900-01;1R01CA224537-01;1R01CA224899-01A1;1R01CA225435-01;1R01CA225446-01;1R01CA225773-01;1R01CA225845-01;1R01CA226527-01;1R01CA226537-01;1R01CA227136-01A1;1R01CA227576-01;1R01CA228512-01;1R01CA228921-01;1R01CA229215-01;1R01CA229415-01;1R01CA229811-01;1R01CA229818-01;1R01CA230323-01;1R01CA230328-01;1R01CA231139-01;1R01CA231325-01;1R01CA234539-01;1R01DA034734-01A1;1R03CA167695-01A1;1R03CA167764-01A1;1R03CA172542-01;1R03CA175462-01A1;1R03CA175889-01;1R03CA176132-01A1;1R03CA182986-01;1R03CA191559-01;1R03CA195147-01;1R03CA202192-01;1R03CA202515-01A1;1R03CA211210-01;1R03CA211831-01;1R03CA211839-01;1R03CA216017-01;1R03CA216142-01A1;1R03CA219621-01;1R03CA222155-01;1R03CA222452-01;1R03CA223731-01A1;1R15CA179409-01A1;1R15CA203605-01;1R15CA219919-01;7R21CA166029-01A1;1R21CA167305-01A1;1R21CA169807-01A1;1R21CA170284-01A1;1R21CA171953-01;1R21CA172938-01A1;1R21CA173271-01A1;1R21CA175974-01;1R21CA175983-01A1;1R21CA177902-01A1;1R21CA178800-01A1;1R21CA181382-01;1R21CA182020-01;1R21CA182725-01A1;1R21CA184282-01;1R21CA184337-01A1;1R21CA184361-01A1;1R21CA185807-01A1;1R21CA185841-01A1;1R21CA186077-01;1R21CA187642-01A1;1R21CA187877-01;1R21CA191158-01A1;1R21CA191651-01A1;1R21CA191751-01;1R21CA194194-01A1;1R21CA194492-01A1;1R21CA195016-01A1;1R21CA195429-01A1;1R21CA196508-01A1;1R21CA197752-01;1R21CA198042-01A1;1R21CA198455-01;1R21CA198462-01A1;1R21CA201567-01A1;1R21CA201963-01;1R21CA202013-01A1;1R21CA202040-01;1R21CA202130-01;1R21CA202263-01A1;1R21CA202417-01A1;1R21CA205778-01;1R21CA205819-01;1R21CA208206-01A1;1R21CA209345-01;1R21CA209875-01A1;1R21CA212386-01A1;1R21CA212687-01A1;1R21CA215252-01A1;1R21CA216772-01A1;1R21CA218592-01;1R21CA219229-01;1R21CA219371-01A1;1R21CA220352-01A1;1R21CA220398-01;1R21CA220670-01;1R21CA223799-01;1R21CA224764-01A1;1R21CA231196-01;1R21CA231214-01;1R21CA231892-01;1R21CA239456-01;1R21CA239457-01;1R33CA206922-01;1R33CA223581-01A1;1R33CA225310-01;1R35CA197627-01;1R37CA214787-01A1;1R37CA222563-01;1R37CA226081-01;1R41CA196565-01;1R41CA224520-01A1;1R43CA176897-01A1;1R43CA183195-01;1R43CA203058-01;1R43CA203455-01;1R43CA228919-01A1;1R43CA232860-01;1R43CA233346-01;1R43CA236164-01;1R44CA228897-01;1U01AI103390-01;1U01CA174706-01;1U01CA176287-01;1U01CA176299-01;1U01CA177799-01;1U01CA179106-01A1;1U01CA180940-01A1;1U01CA180956-01;1U01CA180975-01A1;1U01CA182915-01A1;1U01CA182940-01;1U01CA183081-01;1U01CA184783-01;1U01CA184902-01;1U01CA185188-01A1;1U01CA187947-01A1;1U01CA188383-01;1U01CA188388-01A1;1U01CA195565-01A1;1U01CA195599-01;1U01CA199235-01;1U01CA199252-01;1U01CA199336-01;1U01CA202177-01;1U01CA202958-01;1U01CA206110-01;1U01CA209414-01A1;1U01CA209861-01A1;1U01CA212007-01A1;1U01CA213285-01A1;1U01CA213759-01;1U01CA214114-01;1U01CA214116-01;1U01CA214297-01A1;1U01CA214411-01A1;1U01CA214846-01;1U01CA215709-01A1;1U01CA215798-01;1U01CA215845-01;1U01CA215848-01A1;1U01CA216459-01A1;1U01CA217842-01;1U01CA217846-01;1U01CA217858-01;1U01CA217864-01;1U01CA217885-01;1U01CA220378-01;1U01CA220401-01A1;1U01CA225431-01;1U01CA225451-01;1U01CA225753-01;1U01CA227550-01;1U01CA229437-01;1U01CA229445-01;1U01CA230669-01;1U01CA230694-01;1U01CA231782-01;1U01CA231840-01;1U01CA232137-01;1U01CA232161-01;1U01CA233364-01;1U01CA235487-01;1U01CA235508-01;1U19CA214253-01A1;1UG1CA189805-01;1UG1CA189819-01;1UG1CA189850-01;1UG3CA220642-01;1UG3CA225021-01;1UG3CA233229-01;1UG3CA233251-01;1UG3CA233282-01;1UH2CA203708-01;1UH2CA203711-01;1UH2CA203730-01;1UH2CA203781-01;1UM1CA186705-01;1UM1CA221939-01;1UM1CA221940-01;1UM1CA222035-01;2P01CA154292-06;2R01CA037157-32;2R01CA050286-26A1;2R01CA067850-14;2R01CA096504-11;2R01CA115531-11A1;2R01CA138264-06A1;2R01CA140198-06;2R01CA140657-06;2R01CA151354-06A1;2R01CA163336-06;2R01CA166379-06;2R25CA057712-21;2R42CA189637-02A1;2R42CA203212-02;2R44CA139644-02A1;2R44CA183437-02;2R44CA199058-02;2R44CA213866-02;2U01AI096299-07

The specific aims for these grants were reviewed and data was extracted from the specific aims page for those grants that were considered “Cancer” and “Systems Epidemiology” as described in the “Methods” section.
